# A VCP modulator, KUS121, as a promising therapeutic agent for post-traumatic osteoarthritis

**DOI:** 10.1038/s41598-020-77735-2

**Published:** 2020-11-27

**Authors:** Motoo Saito, Kohei Nishitani, Hanako O. Ikeda, Shigeo Yoshida, Sachiko Iwai, Xiang Ji, Akihiro Nakahata, Akira Ito, Shinichiro Nakamura, Shinichi Kuriyama, Hiroyuki Yoshitomi, Koichi Murata, Tomoki Aoyama, Hiromu Ito, Hiroshi Kuroki, Akira Kakizuka, Shuichi Matsuda

**Affiliations:** 1 Department of Orthopaedic Surgery, Graduate School of Medicine, Kyoto University, Kyoto, Japan; 2 Department of Ophthalmology and Visual Sciences, Graduate School of Medicine, Kyoto University, Kyoto, Japan; 3 Department of Physical Therapy, Human Health Sciences, Graduate School of Medicine, Kyoto University, Kyoto, Japan; 4 Department of Immunology, Graduate School of Medicine, Kyoto University, Kyoto, Japan; 5 Department of Advanced Medicine of Rheumatic Diseases, Graduate School of Medicine, Kyoto University, Kyoto, Japan; 6 Laboratory of Functional Biology, Graduate School of Biostudies, Kyoto University, Kyoto, Japan

**Keywords:** Rheumatology, Musculoskeletal system

## Abstract

Post-traumatic osteoarthritis (PTOA) is a major cause which hinders patients from the recovery after intra-articular injuries or surgeries. Currently, no effective treatment is available. In this study, we showed that inhibition of the acute stage chondrocyte death is a promising strategy to mitigate the development of PTOA. Namely, we examined efficacies of Kyoto University Substance (KUS) 121, a valosin-containing protein modulator, for PTOA as well as its therapeutic mechanisms. In vivo, in a rat PTOA model by cyclic compressive loading, intra-articular treatments of KUS121 significantly improved the modified Mankin scores and reduced damaged-cartilage volumes, as compared to vehicle treatment. Moreover, KUS121 markedly reduced the numbers of TUNEL-, CHOP-, MMP-13-, and ADAMTS-5-positive chondrocytes in the damaged knees. In vitro, KUS121 rescued human articular chondrocytes from tunicamycin-induced cell death, in both monolayer culture and cartilage explants. It also significantly downregulated the protein or gene expression of ER stress markers, proinflammatory cytokines, and extracellular-matrix-degrading enzymes induced by tunicamycin or IL-1β. Collectively, these results demonstrated that KUS121 protected chondrocytes from cell death through the inhibition of excessive ER stress. Therefore, KUS121 would be a new, promising therapeutic agent with a protective effect on the progression of PTOA.

## Introduction

The pathogenesis of osteoarthritis (OA) leads to the degeneration and destruction of articular cartilage and results in significant impairment of joint function and limited mobility in daily life, thereby affecting social activities. There are two types of OA: the primary OA that occurs gradually with ageing but without specific causes, and the secondary OA caused by certain diseases, such as joint injuries, autoimmune inflammatory arthritis, and congenital joint deformities. In post-traumatic osteoarthritis (PTOA), the most common and typical secondary OA, injury to the cartilage or ligament is a well-known cause^1–3^. Despite the advancements in surgical procedures for osteosynthesis and ligament reconstruction over the past few decades, there has been no decrease in the incidence of PTOA, indicating that surgical treatment alone is not sufficient and additional new strategies are necessary to prevent PTOA^4–6^. After a physical damage to the articular cartilage after a traumatic event, acute-stage reactions lead to chondrocyte death followed by matrix damage and induction of proinflammatory cytokines and result in serious and irreversible cartilage degeneration^7–10^. Therefore, it would be beneficial for a new therapeutic agent to target the earliest stage of chondrocyte damage, namely chondrocyte death, to inhibit the progression to PTOA^7,8,11,12^.

Kyoto University Substance (KUS) 121 is a small chemical compound that selectively inhibits the ATPase activity of valosin-containing protein (VCP). VCP belongs to the AAA (ATPases Associated with diverse cellular Activities) family, and is a protein that is universally present in essentially all cells; it is known to play diverse roles, such as proteolysis via the proteasome system in cells, endoplasmic reticulum-associated protein degradation, and membrane fusion^13,14^. KUS121 does not interfere these cellular VCP functions but only suppress its ATP consumption^15^. Although the precise molecular mechanism was not fully elucidated, KUSs suppress intracellular ATP decrease by inhibiting ATPase activities of VCP, and reduces endoplasmic-reticulum (ER) stress, resulting in the inhibition of cell death^15,16^. KUS121 has been shown to protect against cell death in animal models of ocular diseases including retinitis pigmentosa^15,17^, macular degeneration^18^, and glaucoma^19^, ischemic diseases including myocardial infarction^16^, ischemic stroke^20^, and retinal artery occusion^21^ and Parkinson’s disease^22^. Moreover, phase I/II clinical trials show its safety and potential effectiveness in patients with non-arteritic central retinal artery occlusion^23^. ER stress has also been shown to contribute to the degeneration of chondrocytes in the articular cartilage; excessive ER stress induces chondrocyte death in parallel with the severity of OA^24–28^. This knowledge prompted us to test the efficacy of KUS121 on cartilage injury, known as a initial event leading to PTOA.

In the current study, we hypothesized that KUS121 reduces chondrocyte death and inflammatory response in vitro and protects cartilage degeneration in vivo by reducing ER stress in chondrocytes. The aim of this study was to show KUS121 (1) inhibits chondrocyte death following cartilage trauma and thus reduces subsequent cartilage degeneration in a rodent model of cartilage injury, and (2) inhibits chondrocyte death by the suppression of excessive ER stress and subsequent upregulation of pro-inflammatory cytokines in human chondrocytes.

## Results

### KUS121 showed protective effect against cell death and cartilage damage in a rat model of PTOA

**Figure 1:**
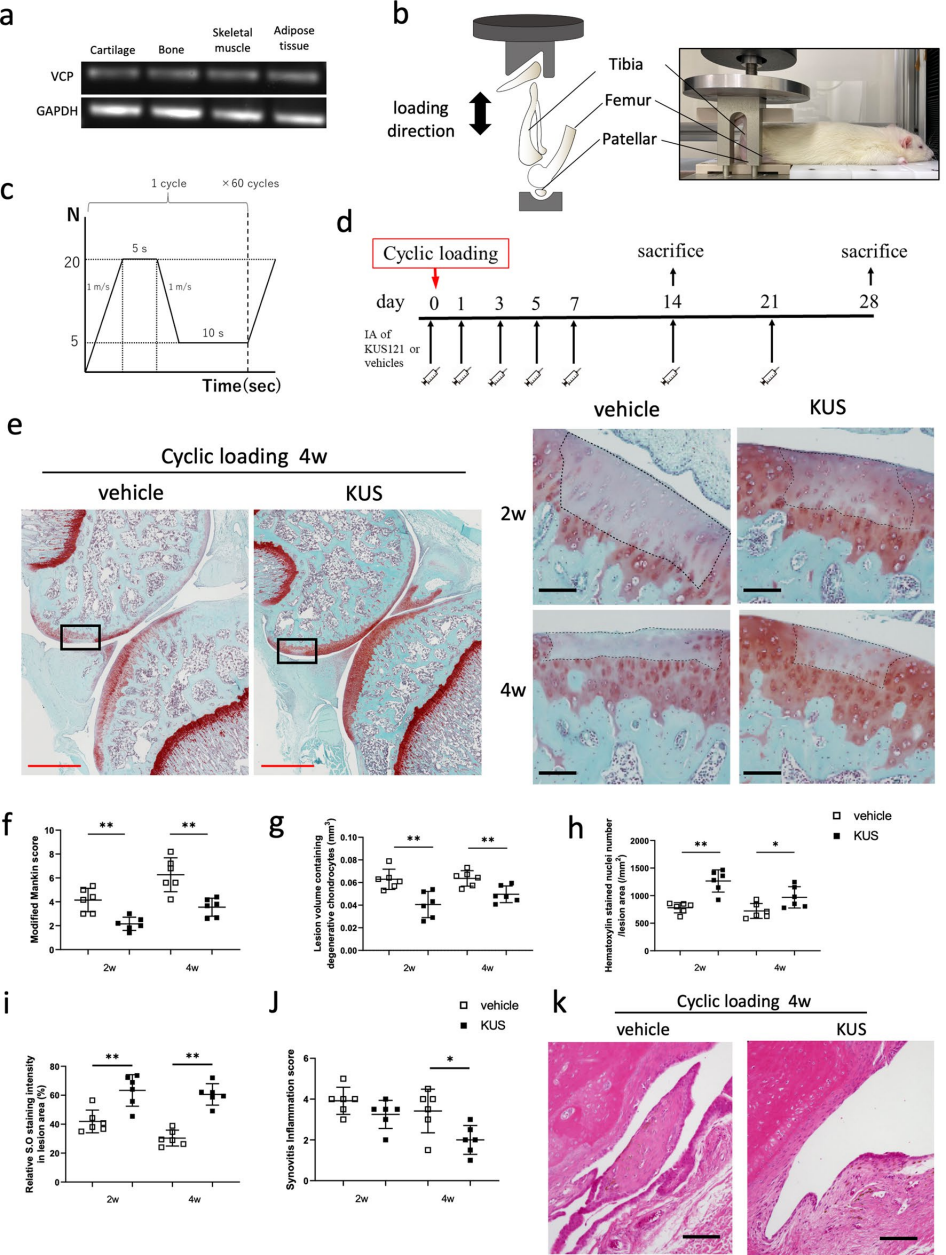
KUS121 attenuates cell death and cartilage damage induced by cyclic compressive loading in rats. (**a**) Representative RT-PCR gel showing expression of VCP and GAPDH in various organ of a Wistar rat. Complete scans of PCR gel are shown in Supplementary Fig. S4; (**b**) Schematic diagram of the non-invasive cyclic compression model. The right knee of an anaesthetised rat was fixed on a customised apparatus with the patella embedded in a loading dent. (**c**) Loading regimen containing 5 s peak load and 10 s rest interval (20 N and 5 N, respectively). (**d**) Experimental protocol of the cyclic compressive loading rat model treated with KUS121 or vehicle. (**e**) Representative safranin-O fast green staining sections in low and high magnification; (**f**) the histological severity of degeneration was evaluated using the modified Mankin scoring system; (**g**) lesion volume and (**h**) haematoxylin-stained nuclei number were evaluated to compare the two groups; (**i**) relative intensity of Safranin O staining in the lesion area, presented in percentage by dividing the intensity in growth plate area; (**j**) synovitis score in the rat knee joints. The area enclosed by the dashed line indicates the cartilage lesion. (**k**) Representative sections of the knee synovium. The black scale bar is 100 μm, and the red bar is 1 mm. Bars in each graph represent the mean ± standard deviation (n = 6 per experimental group and time point). *P < 0.05, **P < 0.01 by unpaired t-test. *IA* intra-articular injection.

**Figure 2:**
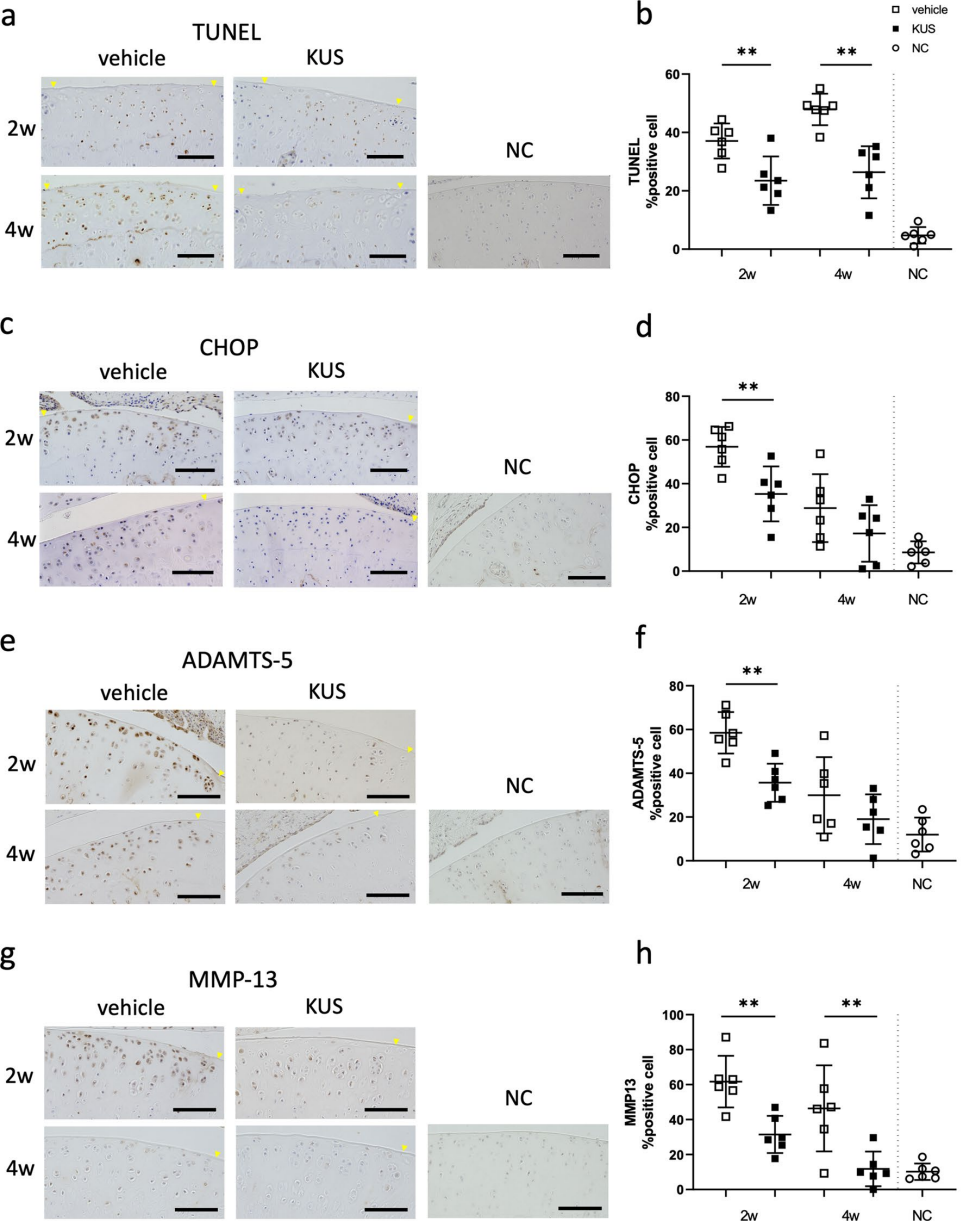
KUS121 attenuates cartilage-matrix proteases and ER-stress markers related to apoptosis induced by cyclic compressive loading in rats. (**a**,**b**) TUNEL staining of the lesion area in each group at 2 and 4 weeks; Immunohistochemical staining of cytoplasmic CHOP (**c**,**d**), ADAMTS-5 (**e**,**f**), and MMP-13 (**g**,**h**), respectively, at 2- and 4-weeks adjacent to the lesion area. The yellow arrowhead indicates the boundary of the cartilage lesion. The scale bar is 100 μm. Bars in each graph represent the mean ± standard deviation (n = 6 per experimental group and time point). *P < 0.05, **P < 0.01 by unpaired-t test. *NC* non-injury control.

We first examined whether the messenger RNA (mRNA) of VCP was ubiquitously expressed in various organs in rat. Expressions of VCP were confirmed in cartilage, bone, skeletal muscle, and subcutaneous adipose tissue in rat (Fig. 1a). Next, the rat PTOA model was generated as described in the “Methods” section (Fig. 1b,c). KUS121 was injected intra-articularly into the knee joint of the rats as shown in the protocol (Fig. 1d). The cartilage lesion was reproducibly produced at the lateral femoral condyle by the cyclic compression (Fig. 1e), and the modified Mankin scores in KUS121-treated rats were significantly lower than those in vehicle-treated rats at 2 and 4 weeks (Fig. 1f). In KUS121-treated rats, the number of haematoxylin-stained live chondrocytes was significantly greater, the lesion volume was significantly smaller, and the intensity of the Safranin O staining at the cartilage lesion was significantly higher than that in the vehicle-treated control rats at both time points (Fig. 1g–i). Mild synovitis was observed in both groups, but the thickening of the lining layer was more conspicuous in vehicle-treated rats (Fig. 1j). The synovitis inflammatory scores in KUS121-treated rats were significantly lower than those in vehicle-treated rats at 4 weeks (Fig. 1k). The number of TUNEL-positive chondrocytes in the articular cartilage lesion was significantly lower in the KUS121-treated than in vehicle-treated rats (Fig. 2a,b), indicating the protective effect of KUS121 against apoptosis. Immunohistochemical analyses revealed that KUS121 significantly decreased the nuclear CHOP-positive chondrocytes in the articular cartilage of experimental rats compared to vehicle-treated rats (Fig. 2c,d). The expression levels of ADAMTS-5 and MMP-13 proteins were also consistently reduced in rats treated with KUS121 (Fig. 2e–h). Likewise, expression levels of ADAMTS-5 and MMP-13 in synovial tissue were attenuated in rats treated with KUS121 (Supplement Fig. S1).

### KUS121 prevent ATP depletion and apoptosis induced by tunicamycin (TM)

**Figure 3:**
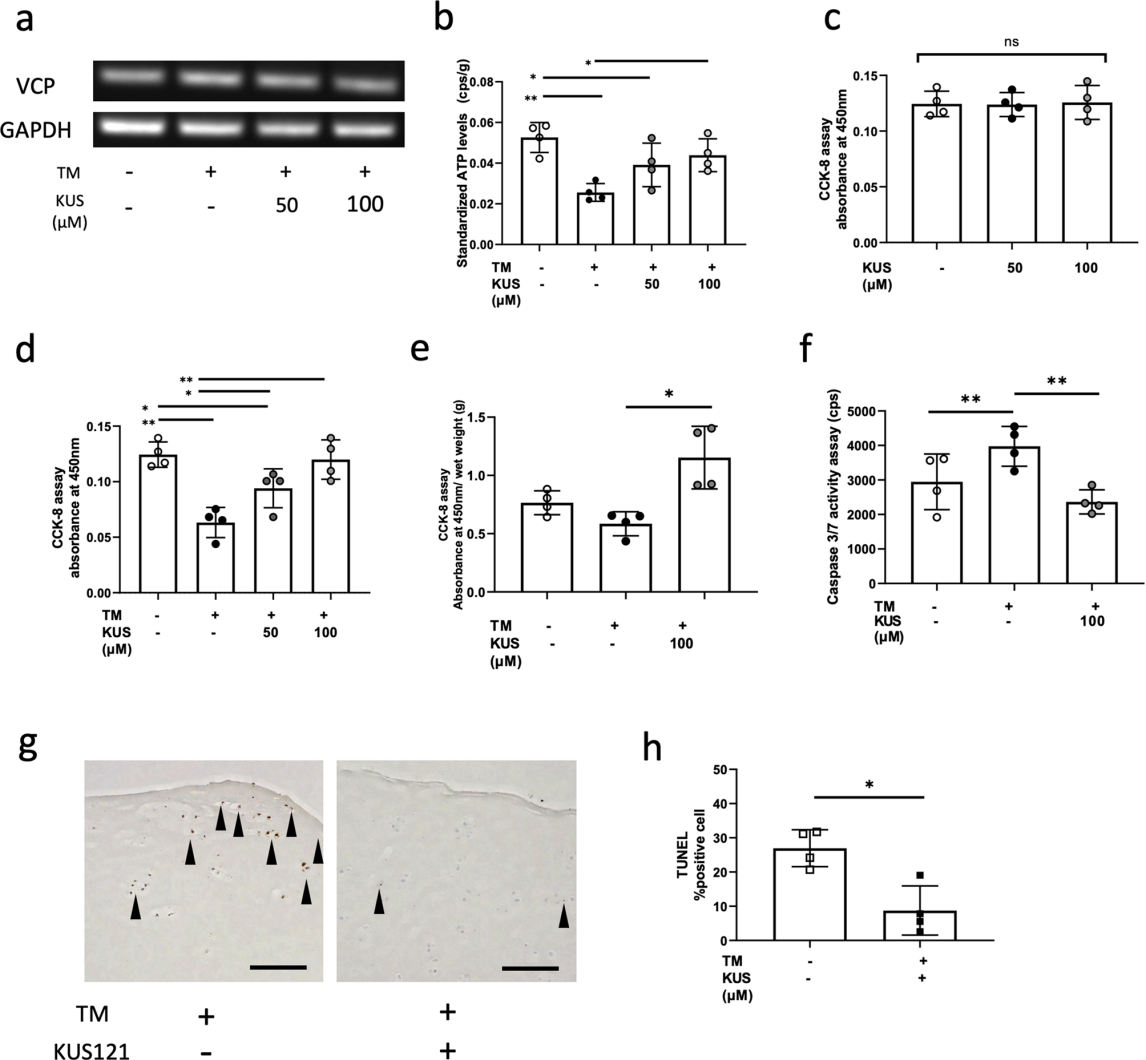
KUS121 prevents ATP depletion and inhibits apoptosis caused by TM. (**a**) Representative PCR gel showing expression of VCP and GAPDH. Complete scans of PCR gel are shown in Supplementary Fig. S4; (**b**) Measurement of the ATP levels. Concentrations of cell lysate in each well were used for standardisation. (**c**) Cytotoxicity against human articular chondrocytes (n = 4) of KUS121 was assessed using Cell Counting kit-8 (CCK-8) assay. Cell viability was also determined with CCK-8 assay using (**d**) human articular chondrocytes with TM at 3 μg/mL or KUS121 at 50 and 100 μM, and using (**e**) human articular cartilage explants (n = 4) with or without TM at 3 μg/mL and KUS121 at 100 μM; (**f**) Caspase 3/7 activity was assessed using fluorometric assay in human articular chondrocytes incubated with or without TM at 3 μg/mL and KUS121 at 100 μM; (**g**,**h**) Representative images of TUNEL staining in human articular cartilage explants stimulated with TM at 10 μg/mL or with KUS121 at 100 μM. Black arrow heads indicate TUNEL-positive cells. Bars represent the mean ± standard deviation (n = 4), *P < 0.05, **P < 0.01 by repeated measured one-way ANOVA with Turkey test except (h) by paired t-test.

We also confirmed that the expression of VCP mRNA in human articular chondrocytes, and its levels were not affected by the TM or KUS121 treatment (Fig. 3a). However, it is notable that treatment with 50 μM and 100 μM KUS121 marginally and significantly recovered the ATP levels reduced by TM, respectively (Fig. 3b). The protective effect of KUS121 against human articular chondrocytes cell death was investigated using the Cell Counting Kit (CCK-8) assay. KUS121 had no effect on human chondrocyte viability (Fig. 3c). On the other hand, incubation with 10 µg/mL TM decreased cell viability, but a simultaneous incubation with KUS121 significantly recovered chondrocyte viability (Fig. 3d). Human-cartilage explants treated with KUS121 with or without TM for 48 h also showed survival effects of KUS121 on chondrocytes existing in the cartilage matrix (Fig. 3e). KUS121 significantly reduced the activation of caspase-3 and -7 in TM-treated chondrocytes (Fig. 3f). Additionally, KUS121 treatment showed a significant decrease in the number of TUNEL-positive cells in TM-treated human cartilage explants compared to those without KUS121 treatment (Fig. 3g,h).

### KUS121 alleviated TM-induced ER stress in chondrocytes

**Figure 4:**
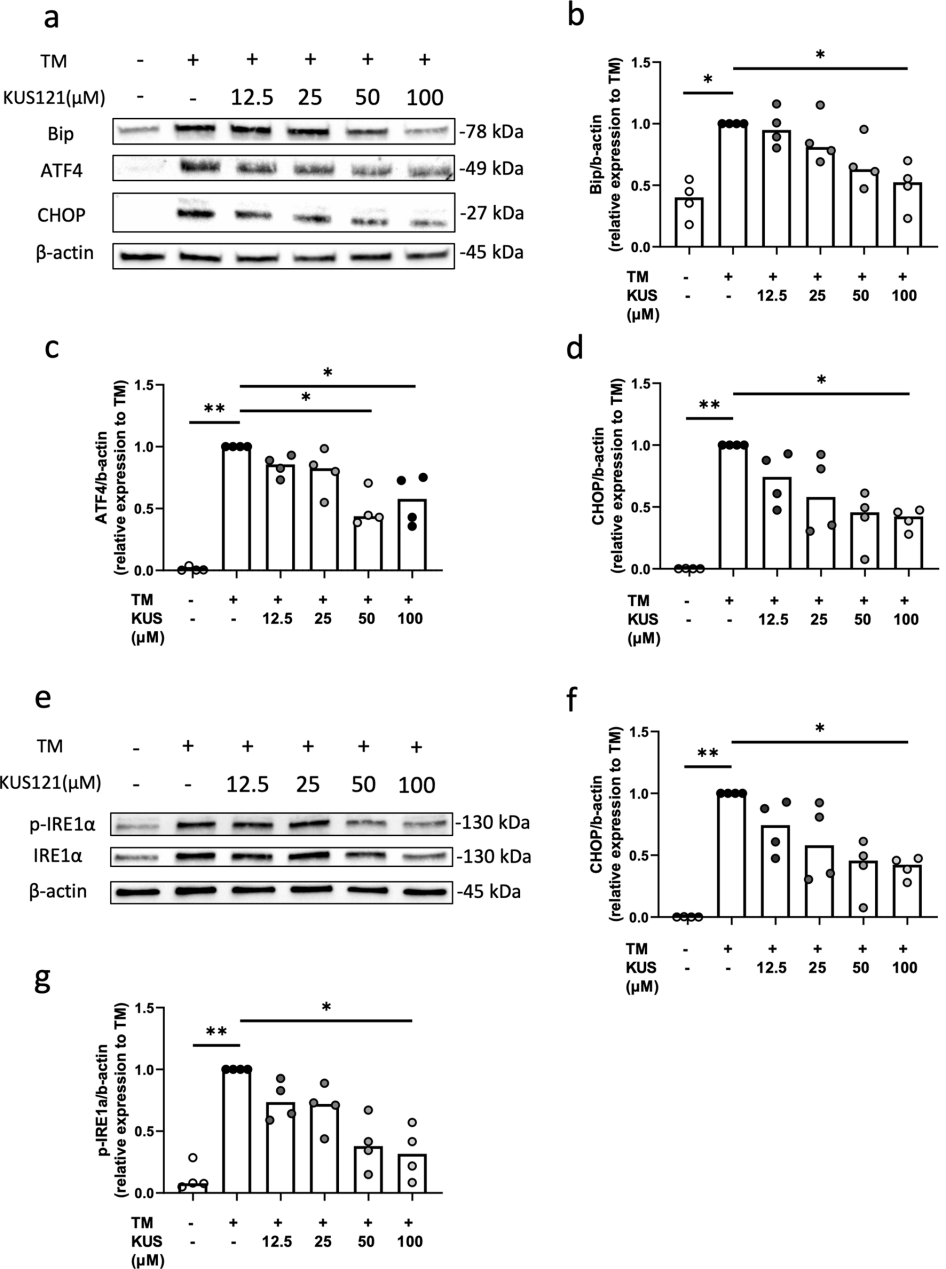
KUS121 inhibits the expression of the mediators of ER stress-induced apoptosis protein in human chondrocytes. (**a**) Representative immunoblots of Bip, ATF4, and CHOP. Immunoblotting of β-actin was used as internal control for total proteins. Complete scans of western blots are shown in Supplementary Fig. S5; (**b**–**d**) Cumulative data of Bip, ATF4, and CHOP after densitometry quantification of immunoblots; (**e**) Representative immunoblots of IRE1α and p-IRE1α. Complete scans of western blots are shown in Supplementary Fig. S6; (**g**,**f**) Cumulative data of IRE1α and p-IRE1α, respectively, after densitometry quantification of immunoblots. Chondrocytes were treated with TM at 3 μg/mL or with 12.5, 25, 50, and 100 μM KUS121 for 8 h. Bars represent the median (n = 4), *P < 0.05, **P < 0.01 to TM (second column) by Friedman test with Dunn’s post-test.

**Figure 5:**
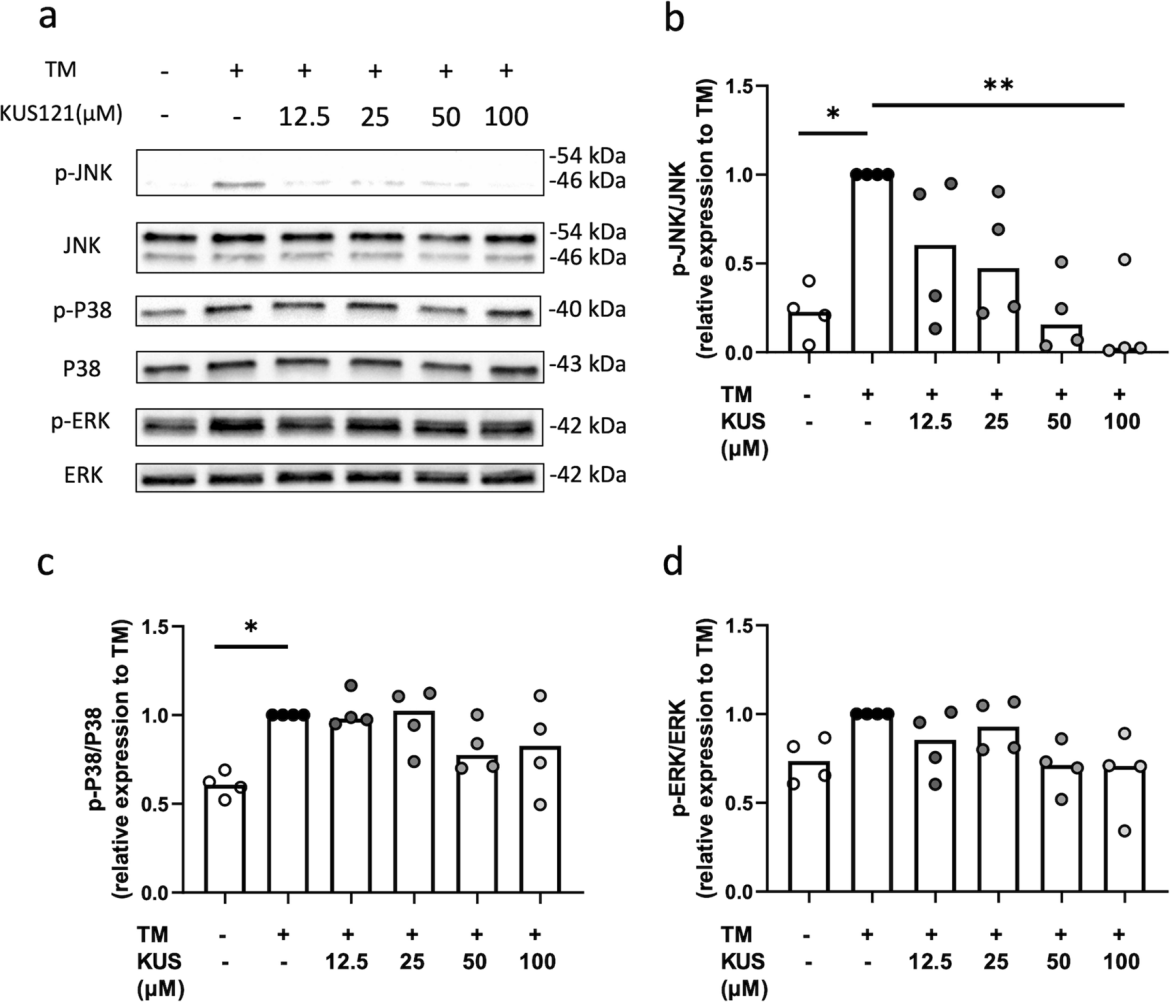
KUS121 inhibits the activation of JNK / stress-activated protein kinases derived from ER stress. (**a**) Representative immunoblots of JNK, p-JNK, p38, and p-p38. β-actin was used as internal control for total proteins. Complete scans of western blots are shown in Supplementary Fig. S7 (**b**–**d**) Cumulative data for p-JNK, p-p38, and p-ERK, respectively, after densitometry quantification of immunoblots. Chondrocytes were treated with TM at 3 μg/mL or with 12.5, 25, 50, and 100 μM KUS121 for 8 h. Bars represent the median (n = 4), *P < 0.05, **P < 0.01 to TM (second column) by Friedman test with Dunn’s post-test.

Protein levels of Bip (an ER-stress marker) and ATF4 and CHOP (markers of ER stress-induced apoptosis) were examined. KUS121 attenuated the upregulation of these proteins (Fig. 4a–d). Moreover, protein and phosphorylation levels of another ER-stress marker, inositol-requiring enzyme 1 α (IRE1α), in TM-treated samples were also significantly attenuated by KUS121 (Fig. 4e–g). KUS121, but not the vehicle treatment, reduced the protein and phosphorylation levels of JNK, existing in another ER stress-induced apoptosis pathway downstream of IRE1α, in TM-treated chondrocytes (Fig. 5a,b). However, phosphorylation levels of ERK and P38 belonging to the mitogen-activated protein kinase (MAPK) family were not affected by KUS121 (Fig. 5a,c,d).

### KUS121 decreased pro-inflammatory cytokine and extracellular matrix (ECM) catabolic proteases in chondrocytes

**Figure 6:**
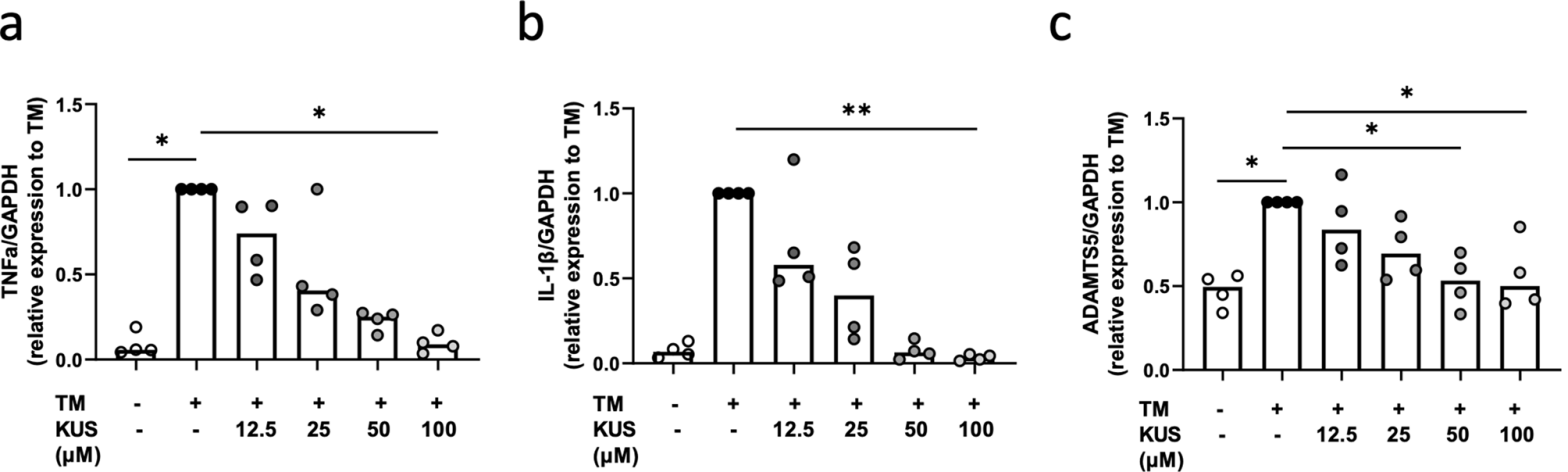
KUS121 suppresses the expression of TM-induced proinflammatory cytokines and proteases. Transcriptional levels of (**a**) TNF-α, (**b**) IL-1β, and (**c**) ADAMTS5 in chondrocytes cultured with TM at 10 μg/mL or with KUS121 at 12.5, 25, 50, and 100 μM for 4 h were determined using reverse-transcription quantitative polymerase chain reaction. RNA expression levels were normalised relative to the expression of GAPDH. Bars represent the median (n = 4), *P < 0.05, **P < 0.01 to TM (second column) by Friedman test with Dunn’s post-test.

**Figure 7:**
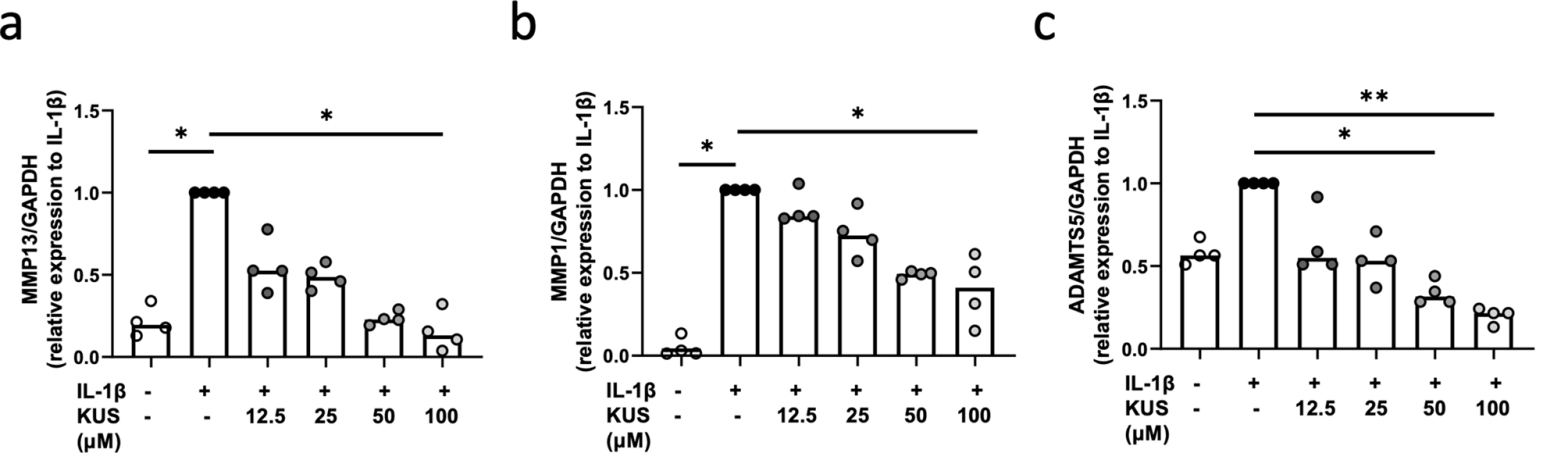
KUS121 suppresses the expression of IL-1β-induced cartilage-matrix proteases. Transcriptional levels of (**a**) MMP13, (**b**) MMP1, and (**c**) ADAMTS5 in chondrocytes cultured with IL-1β at 2 ng/mL or with 12.5, 25, 50, and 100 μM KUS121 for 12 h were determined using reverse-transcription quantitative polymerase chain reaction. RNA expression levels were normalised relative to the expression of GAPDH. Bars represent the median (n = 4), *P < 0.05, **P < 0.01 to IL-1β (second column) by Friedman test with Dunn’s post-test.

Expression levels of TNF-α and IL-1β mRNAs in human articular chondrocytes induced by TM were significantly suppressed with KUS121 (Fig. 6a,b). Expression levels of ADAMTS5 mRNA were also significantly attenuated by KUS121. As compared with TNF-α and IL-1β mRNAs, however, the overall degree of attenuation was lower (Fig. 6c). Note that the expression of MMP-1 and MMP-13 mRNA was not upregulated by TM at a dose of 10 μg/mL (data not shown). However, treatment with 2 ng/mL IL-1β dramatically induced the expression of MMP-1 and MMP-13 mRNA (Fig. 7a,b). Treatment with 2 ng/mL IL-1β also induced the mRNA expression of ADAMTS5, but to a lesser extent as compared with those of MMP-1 and MMP-13 (Fig. 7c). These upregulated expressions were attenuated by KUS 121(Fig. 7a–c). In contrast, neither TM nor IL-1β and KUS121 influenced the expression levels of col2a1, aggrecan, and SOX9 mRNAs (Supplementary Figs. S2, S3).

## Discussion

While primary OA occurs gradually with an increase in age without any apparent causes, secondary OA has causes, in which intra-articular injuries are the most common and typical (referred to as PTOA). There are currently no disease-modifying drugs available for any type of OA. Thus, the very early period after injury may be the only chance to intervene to decrease the cartilage damage which may lead to PTOA. However, there are no available drug to prevent such early lesion, either. Therefore, we examined whether KUS121, our new compound, was able to prevent it. In this study, we reported that KUS121 was able to protect TM-treated cultured chondrocytes from death by reducing ER stress. In the acute phase of cartilage trauma, chondrocyte death is followed by catabolic effects in the intra-articular tissues, owing to inflammatory mediators such as proinflammatory cytokines, chemokines, and damage-associated molecular patterns. Thus, the suppression of such mediators potentially reduces the onset of PTOA^29,30^.

In this study, direct-knee injection of KUS121 was found to be efficacious in reducing cartilage lesion volumes, suppressing degenerative changes of the cartilage, and decreasing cell death in lesions, as well as supressing expressions of ECM-degrading protease, all of which are seen in very early stages of OA. As the mechanism, we demonstrated that KUS121 dramatically reduced the production of pro-inflammatory cytokines such as TNF-α and IL-1β, and also significantly inhibited the expression of chondrocyte proteases such as MMPs and ADAMTS5 that are responsible for the breakdown of the ECM in chondrocytes. It is notable that there was no persistent elevation of these protease expressions in cartilage and synovium at 4 weeks. If there were continuous stress in the joint by ligament tear or meniscus instability, these protease expressions would persist, but probably due to the lack of continuous stress on the joint in our model, these expressions subsid, similar to human cartilage injury without ligamentous tear. Pro-inflammatory cytokines are important disease modifying factors for the onset of OA as well as during the course of the progression of PTOA. However, we were not able to obtain the data showing IL-1β alone induced cartilage cell death (data not shown). Instead, tunicamycin was able to reproducibly induce ER stress, cell death, and following elevation of inflammatory cytokines. We, therefore, used tunicamycin as an inducer of disease-related phenotypes.

Previous reports show that ER stress-induced apoptosis is significantly involved in the pathogenesis of OA. Takada et al.^24^ report enhanced expression of CHOP and p-JNK in severely degenerated cartilage tissue compared to that in mildly or moderately degenerated cartilage tissue. Uehara et al.^25^ report that cartilage degeneration is attenuated with reduced expression of the apoptosis-execution caspase-3 in CHOP-knockout mice. Consistent with genetic studies, we demonstrated that KUS121 decreased CHOP and p-JNK expression by suppressing ER stress and consequently protected chondrocytes against cell death. It has been shown in a mouse model of arthritis that ER stress provokes inflammation via expression of the ER stress sensor IRE1α that is required for IL-1β activity^31^. Notably, KUS121 did not affect phosphorylation levels of IRE1α; however, it reduced the expression of the IRE1α protein itself that has been shown to be induced by the PERK-ATF4 pathway^32^.

KUS121 is a small chemical compound that selectively inhibits the ATPase activity of VCP^15^. Namely, KUS121 does not interfere with essential functions of VCP such as proteolysis via the proteasome system in cells, ER-associated degradation, and membrane fusion^13,14^, and only suppresses ATP consumption. Thus far, profound relations of ATP decrease, ER stress, and cell death have been repeatedly shown in several cell death-inducing cell culture conditions in various cell types, and KUS121 administration mitigated these three phenomena. Consistent with the present study, administration of KUS121 has been shown to mitigate pathologies by reducing ER stress and cell death in several animal models representing conditions such as retinitis pigmentosa^15^, myocardial infarction^16^, ischaemic stroke^20^, and Parkinson’s disease^22^. Moreover, KUS121 has been tested in phase I/II clinical trial for acute central retinal artery occlusion and shown to be safe and efficacious^23^, thereby indicating its promising candidature in the clinical therapy to inhibit the development of PTOA following cartilage injury and other conditions that have been discussed earlier.

This study has several limitations. First, the cyclic loading model used in this study in rat is not able to produce PTOA-like changes in the whole joint^33^. Although the current animal model was adequate to achieve the purpose of this study to examine protective effect of KUS121 on the suppression of acute chondrocyte death, one of the crucial early events in OA, other OA models would be needed to evaluate the effect of KUS121 on whole joint OA changes. Second, the observation periods our animal models were set to 2 and 4 weeks; long-term observations are needed to evaluate the long-term therapeutic effect of KUS121 on the cartilage lesion. Third, to obtain the maximum effect of KUS121, the drug was administered by articular injection before and multiple times after traumatic intervention. Further investigation is needed to determine the ideal injection regimen to minimise the number of injections. Fourth, paracrine effects of other tissues in the joint was not evaluated. Lastly, the pharmacokinetics of KUS121 post-injection has not been determined and this warrants further investigation.

In conclusion, we demonstrated that KUS121, a VCP modulator, protected articular chondrocytes against cell death by reducing ER stress and the expression of proteases that aggravate OA in cultured chondrocytes. KUS121 decreased cartilage damage by suppressing chondrocyte death, ER stress, and protease expression in a rat model of PTOA. These results indicated the potential of KUS121 as a novel therapeutic agent to treat cartilage injuries leading to secondary OA.

## Methods

### Ethical statement

For the usage of human samples, this study was approved by Kyoto University Graduate School and Faculty of Medicine, Ethics Committee (G0502-1), and written informed consent was obtained from all patients. All methods were performed in accordance with the Declaration of Helsinki and the guidelines from the Ethics Committee. All animal experimental protocols were approved by the Animal Research Committee, Graduate School of Medicine, Kyoto University (MedKyo18253, MedKyo19266). All procedures were performed in accordance with the regulations and guidelines for animal experimentation from the Animal Research Committee of Kyoto University. We complied with the ARRIVE guidelines for reporting animal experiments.

### Rat model of post traumatic osteoarthritis induced by cyclic compressive load

Eleven-week-old male Wistar rats purchased from Japan SLC (Hamamatsu, Japan) were acclimatised to the new environment for a week before interventions. The rats had free access to water and standard rodent chow. A constant 12-h light/dark cycle was maintained. Rats were intraperitoneally administered a combination anaesthetic containing 0.375 mg/kg medetomidine, 2 mg/kg midazolam, and 2.5 mg/kg butorphanol, and were randomly assigned into vehicle- or KUS121-treated groups (6 rats per group). The cartilage injury was exposed by cyclic compression as previously reported^33^. Briefly, each right knee was placed in a prone position on a custom-made table with the knee at approximately 140° flexion and subjected to cyclic compressive loading including a pre-load of 5 N, peak load of 20 N with an approaching speed of 1 mm/s, and a 10 s rest interval. Each session was repeated 60 cycles and lasted approximately 20 min. In preliminary experiments, no ACL tear was observed confirmed under cyclic loading at a peak load of 20 N (n = 5; data not shown). If ACL injury occurred, which was recognized as a sudden pop in the cyclic loading, experiments were terminated. Immediately before cyclic compressive loading and on days 1, 3, 5, 7, 14, and 21 after loading, 100 µL of test formulations were injected into the articular cavity of the right knee along the patella tendon using a 27 G syringe under the isoflurane inhalation anesthesia. The control group was applied with a vehicle (5% glucose solution), and the KUS121-treated group was applied with KUS121 dissolved in 5% glucose solution (20 mg/mL).

### Histology

Rat knee-joint samples and human-cartilage samples were fixed with 4% paraformaldehyde for 24 h at 4 °C. The rat knee-joint samples were decalcified using 10% ethylenediaminetetraacetic acid disodium salt dihydrate (pH 7.4) for 3 weeks at 4 °C. All samples were embedded in paraffin and sectioned to a thickness of 5 μm. To compare the cartilage lesions at the lateral femoral condyle, 5 Safranin O-stained sections per joint were graded based on the modified Mankin scoring system by two blinded observers^34,35^. The damaged cartilage-lesion volume was calculated by integrating the area of the lesion of diminished Safranin-O staining over 10 sections every 50 μm using Image J software (National Institutes of Health, Bethesda, MD, USA). To assess the number of chondrocytes in the cartilage lesion, the haematoxylin-stained cells were counted and standardised by the area of the cartilage lesion of each section; the means over 10 sections were calculated for every 50 μm. To assess the intensity of Safranin O staining at the cartilage lesions, the RGB images of each section were split to Red (R), Green (G) and Blue (B) channels using ImageJ software. Among them, the Red images inverted 8-bit grayscale, and the relative intensity in lesion area was calculated by dividing by the intensity in growth plate^33,36^. To evaluate synovitis in the knee joints, the Haematoxylin and eosin stained sections were graded according to the synovitis inflammatory score as previously reported by Jackson et al.^37^. Briefly, the anterior and posterior regions of the joint were respectively scored at a single section adjacent to the intercondylar fossa, and the average score of each section was calculated. To detect apoptotic cells, TdT-mediated dUTP nick-end-labelling (TUNEL) staining was performed using an in situ Apoptosis Detection kit (Takara Bio, Kusatsu, Japan). The number of TUNEL-positive apoptotic chondrocytes was counted in 3 different sections per knee and expressed as a percentage of total chondrocytes. For immunohistochemistry, sections were immunostained using the VECTASTAIN Elite ABC kit (Vector Laboratories, Burlingame, CA, USA) according to the manufacturer’s protocol. Sample sections treated with primary antibodies (Supplementary Table S2) and the biotinylated secondary antibody were visualised using 3,3′-diaminobenzidine (DAB) substrate (Vector Laboratories) and counterstained with haematoxylin. The number of positive-staining chondrocytes in the region adjacent to the cartilage lesion were counted in four fields in 2 different sections per knee under high-magnification imaging and expressed as a percentage of total chondrocytes.

### Human chondrocyte monolayer culture and cartilage explant culture

Human articular chondrocytes were harvested from patients (13 females and 7 males, mean age ± standard deviation: 71.5 ± 3.8 years old, minimum: 65 years old, maximum: 79 years old) with primary OA who underwent total knee arthroplasty. Preparation and isolation of primary human articular chondrocytes were conducted as previously reported^38^. In brief, cartilages taken from the area corresponding to the classification of International Cartilage Repair Society (ICRS) grade 0 or 1 were minced and digested using 0.25% trypsin–EDTA (Thermo Fisher Scientific, Waltham, MA, USA) at 37 °C for 30 min followed by a 24-h incubation in 4 mg/mL collagenase (Wako, Osaka, Japan). Chondrocytes were then cultured in DMEM/F-12 containing 10% foetal bovine serum with 100 U/mL penicillin and 100 mg/mL streptomycin in 35 mm 6-well plates or 96-well (Corning, Corning, NY, USA) at 37 °C in a humidified 5% CO_2_ atmosphere until 80% confluency. Serum-starved primary chondrocytes were treated with KUS121 at 12.5, 25, 50, or 100 µM, and 3 µg/mL tunicamycin (Cayman Chemical, Ann Arbor, MI, USA), or recombinant IL-1β at 2 ng/mL (Sigma-Aldrich, Saint Louis, MO, USA). The fresh full-depth cartilage plug (ϕ 5 mm) explants were harvested from the area of ICRS grade 0, maintained in serum-free DMEM for 48 h at 37 °C, then treated with TM (10 µg/mL) with or without KUS121 (100 µM) for 24 h at 37 °C. Wet weights of each explant cartilage were measured for standardisation. Four biological replicates were performed per each experiment using human samples**.**

### Cell viability assay

Cell viability was evaluated using the Cell Counting Kit-8 (CCK-8) assay (Dojindo Laboratories, Kumamoto, Japan); the caspase 3/7 activity assay was used to determine caspase activity in human chondrocytes using the Caspase-Glo 3/7 assay (Promega, Madison, WI, USA) according to the manufacturer’s protocol.

### ATP assay

ATP levels in human chondrocyte lysates were determined using a luciferase chemiluminescence-based ATP assay for cells (Toyo B-net, Tokyo, Japan) following the manufacturer’s protocol. Briefly, cell lysis buffer, provided by the manufacture, was added to each well of cultured chondrocytes, and luminescence signal was measured after 5 min of incubation at room temperature. The number of cells in each well was standardized by protein concentration, measured using the Pierce BCA Protein Assay kit (Thermo Fisher Scientific).

### Determination of mRNA expression

Total RNA from primary human chondrocytes and rat tissues (cartilage, bone, muscle and adipose tissue) was purified using an RNeasy Mini Kit (Qiagen, Hilden, Germany) followed by reverse transcription using ReverTra Ace qPCR RT Master Mix (TOYOBO, Osaka, Japan) according to the manufacturer’s protocol. Then, SYBR Green real-time PCR (TOYOBO) was performed using Step One Plus (Thermo Fisher Scientific) in triplicate for each sample to determine relative gene expression using glyceraldehyde-3-phosphate dehydrogenase as a housekeeping control using the 2^−ΔΔCt^ method. To confirm the expression of VCP, the PCR products were electrophoresed on 2% agarose gels containing ethidium bromide, and detected using ChemiDoc Plus (Bio-Rad, Hercules, CA, USA). Primer sequences are shown in Supplementary Table S1.

### Western blotting

The chondrocytes were lysed in RIPA buffer containing a protease- and phosphatase-inhibitor cocktail (Nacalai Tesque, Kyoto, Japan). Total protein concentrations were measured using the Pierce BCA Protein Assay kit (Thermo Fisher Scientific). Equal amounts of protein samples were fractionated using SDS-PAGE gels (4–20%) and then electro-transferred to polyvinylidene difluoride (PDVF) membranes. The membranes were blocked using Blocking One (Nacalai Tesque) for 40 min at room temperature followed by overnight incubation at 4 ℃ with primary antibodies (see Supplementary Table S2), and then with an HRP-linked secondary antibody (Cell Signaling Technology, Danvers, MA, USA) for 1 h at room temperature. Immunoreactive proteins were detected with ECL Plus (Cytiva, Marlborough, MA, USA) using ChemiDoc Plus (Bio-Rad).

### Statistics

The results are presented as dot plots and mean ± standard deviation in normally distributed data, and as dot plots with median in non-normally distributed data. Statistical comparisons of the data between two groups were conducted using unpaired student’s *t* test or paired-t test. For three or more groups, non-parametric Friedman test was used for non-normally distributed data, and one-way ANOVA with Tukey’s multiple comparison post-test was performed for normally distributed data. Statistical analyses were performed using GraphPad Prism version 7.00 (GraphPad Software, San Diego, CA, USA). *P* < 0.05 was considered statistically significant.

## Supplementary information

Supplementary Information.

## Data Availability

The datasets analysed during the current study available from the corresponding author on reasonable request.
