# Author Correction: A VCP modulator, KUS121, as a promising therapeutic agent for post-traumatic osteoarthritis

**DOI:** 10.1038/s41598-021-86883-y

**Published:** 2021-03-29

**Authors:** Motoo Saito, Kohei Nishitani, Hanako O. Ikeda, Shigeo Yoshida, Sachiko Iwai, Xiang Ji, Akihiro Nakahata, Akira Ito, Shinichiro Nakamura, Shinichi Kuriyama, Hiroyuki Yoshitomi, Koichi Murata, Tomoki Aoyama, Hiromu Ito, Hiroshi Kuroki, Akira Kakizuka, Shuichi Matsuda

**Affiliations:** 1 Department of Orthopaedic Surgery, Graduate School of Medicine, Kyoto University, Kyoto, Japan; 2 Department of Ophthalmology and Visual Sciences, Graduate School of Medicine, Kyoto University, Kyoto, Japan; 3 Department of Physical Therapy, Human Health Sciences, Graduate School of Medicine, Kyoto University, Kyoto, Japan; 4 Department of Immunology, Graduate School of Medicine, Kyoto University, Kyoto, Japan; 5 Department of Advanced Medicine of Rheumatic Diseases, Graduate School of Medicine, Kyoto University, Kyoto, Japan; 6 Laboratory of Functional Biology, Graduate School of Biostudies, Kyoto University, Kyoto, Japan

Correction to: *Scientific Reports* 10.1038/s41598-020-77735-2, published online 27 November 2020

This Article contains an error in Figure 4, where Figure 4f is a duplication of Figure 4d. The correct Figure 4 appears below.

**Figure 4:**
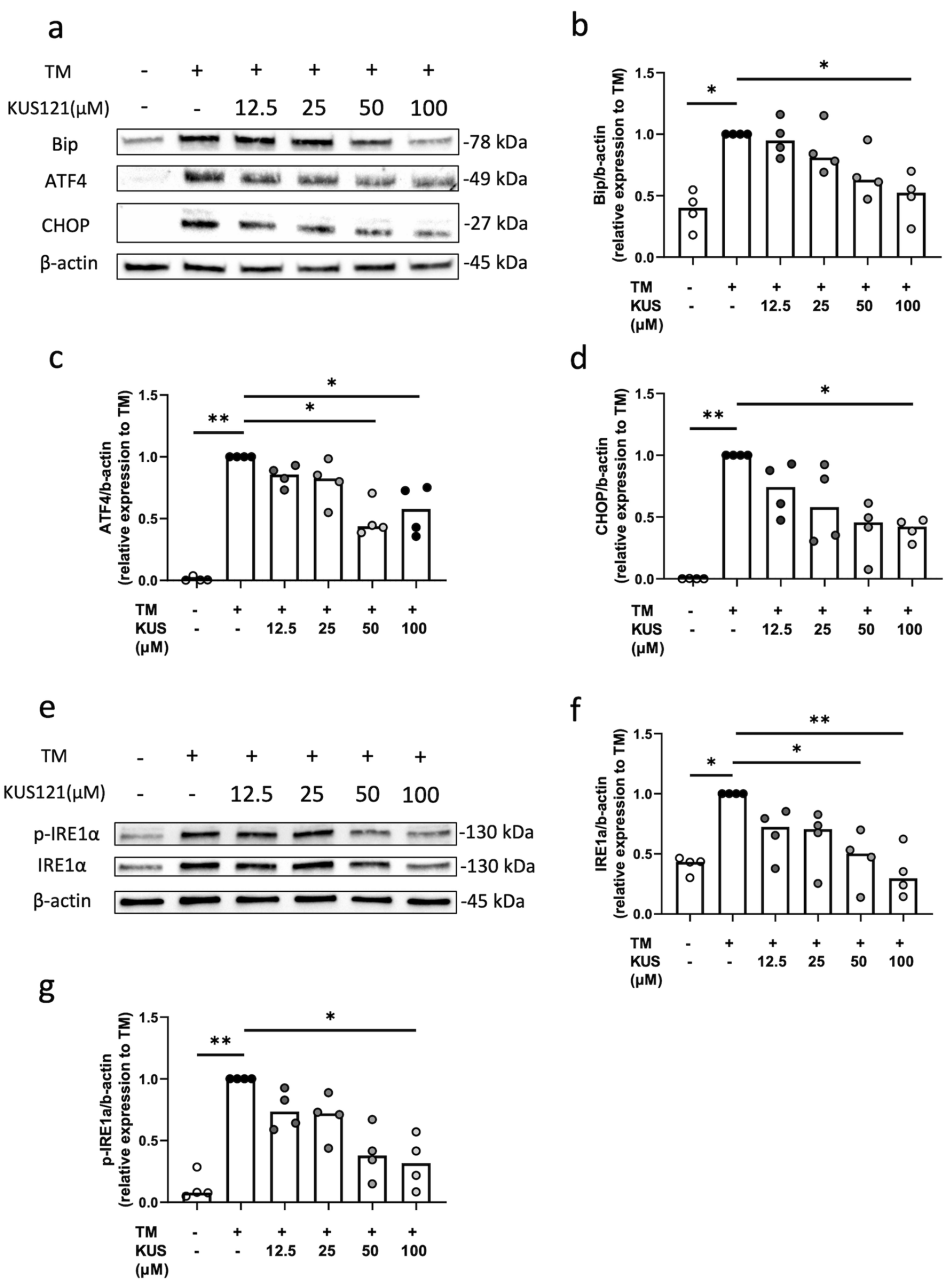
A correct version of the original Figure 4.

