# Supplementary material for: A VCP modulator, KUS121, as a promising therapeutic agent for post-traumatic osteoarthritis
Source: Sci Rep. 2020 Nov 27;10:20787. doi: 10.1038/s41598-020-77735-2 (PMC7695735; doi:10.1038/s41598-020-77735-2)
Supplement: Supplementary file 1 — Supplementary Information. [file 41598_2020_77735_MOESM1_ESM.pdf]

Supplementary information

A VCP Modulator, KUS121, as a Promising Therapeutic Agent for Post-Traumatic Osteoarthritis

Motoo Saito, Kohei Nishitani, Hanako O Ikeda, Shigeo Yoshida, Sachiko Iwai, Xiang Ji, Akihiro Nakahata, Akira Ito, Shinichiro Nakamura, Shinichi Kuriyama, Hiroyuki Yoshitomi, Koichi Murata, Tomoki Aoyama, Hiromu Ito, Hiroshi Kuroki, Akira Kakizuka, Shuichi Matsuda

Supplementary Fig. S1.

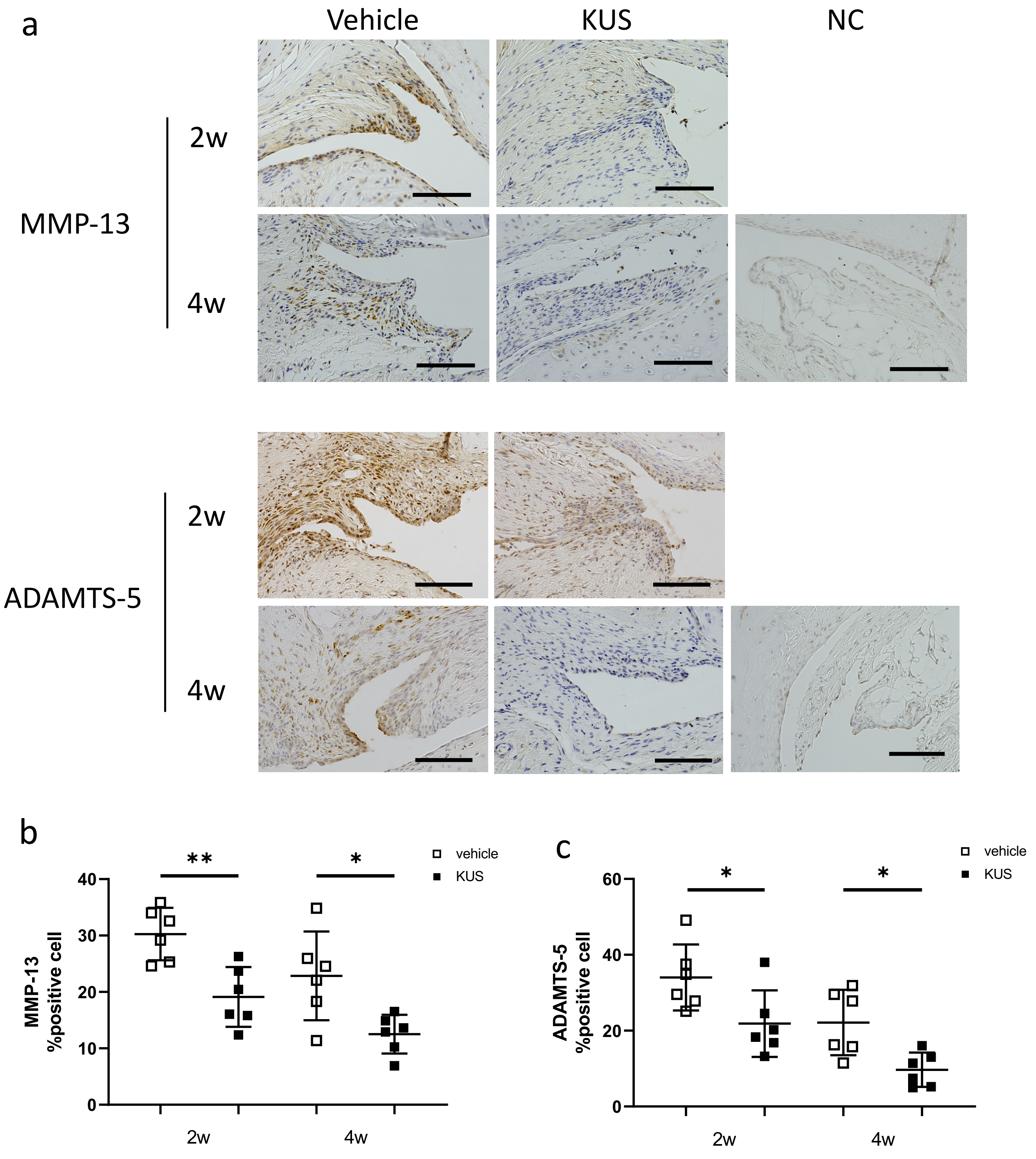

Supplementary Figure S1. (a) Immunohistochemical staining of MMP13 (upper) and ADAMTS5 (lower) at 2- and 4-weeks in the synovium of lateral posterior femoral recess. (b, c) The percentage of positive cells of MMP-13 (b) and ADAMTS-5 (c). The black scale bar is 100 μm. Bars in each graph represent the mean ± standard deviation (n = 6 per experimental group and time point). * P<0.05, ** P<0.01 by unpaired t-test. NC: negative control

Supplementary Fig. S2.

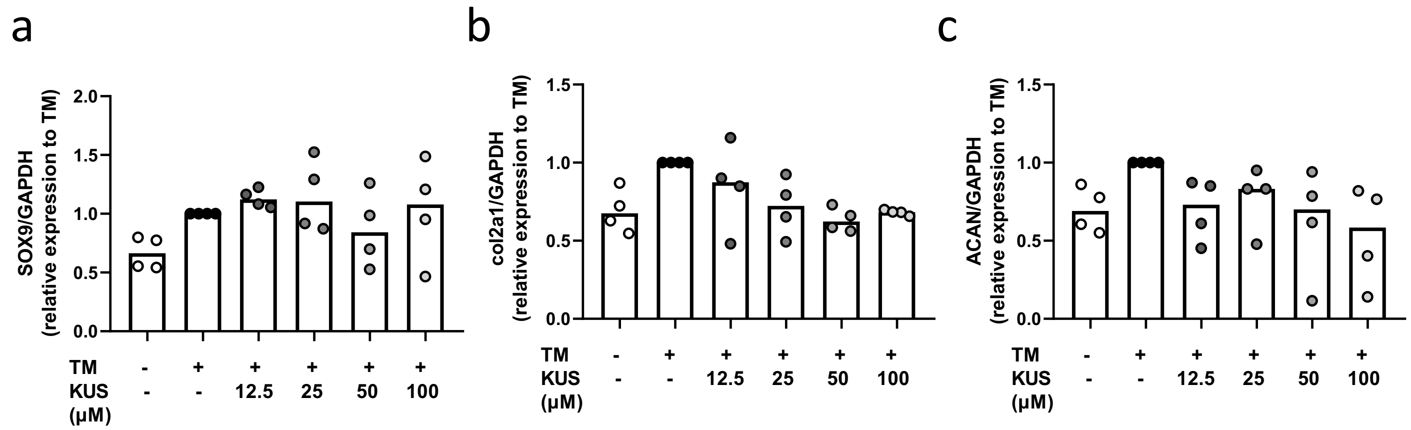

Supplementary Fig S2. Transcriptional levels of (a) SOX9, (b) col2a1,and (c) ACAN in chondrocytes cultured with TM at10μg/ml or with each concentration of KUS121 (12.5μM, 25μM, 50μM, 100μM) for 4 hr were analyzed using reverse transcription-quantitative polymerase chain reaction analysis. RNA expression levels were normalized relative to the expression of GAPDH. Bars represent the median ± interquartile range (n=4).

Supplementary Fig. S3.

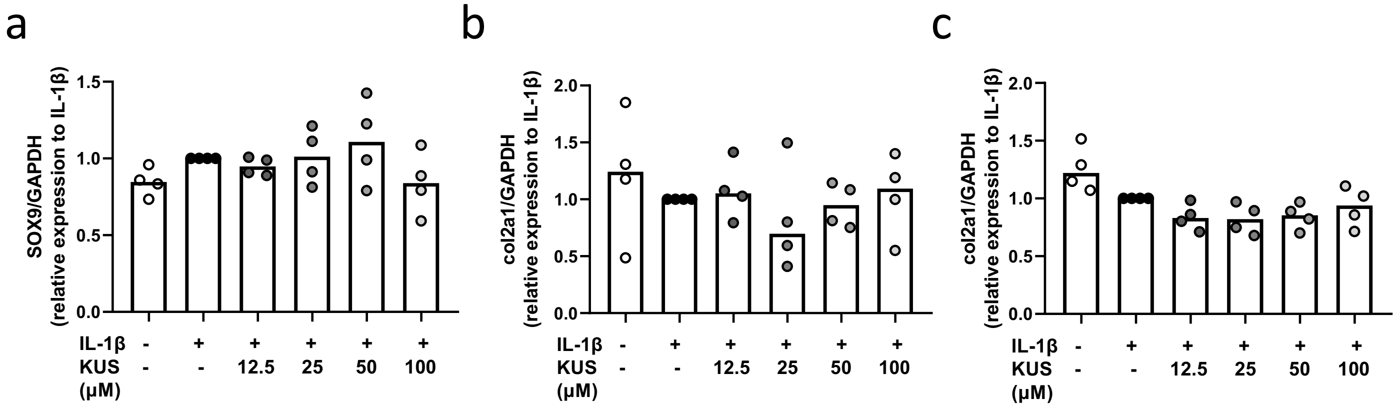

Supplementary Fig S3. Transcriptional levels of (a) SOX9, (b) col2a1,and (c) ACAN in chondrocytes cultured with IL-1ß at 2ng/ml or with each concentration of KUS121 (12.5μM, 25μM, 50μM, 100μM) were analyzed using reverse transcription-quantitative polymerase chain reaction analysis. RNA expression levels were normalized relative to the expression of GAPDH. Bars represent the median (n=4).

Supplementary Fig. S4.

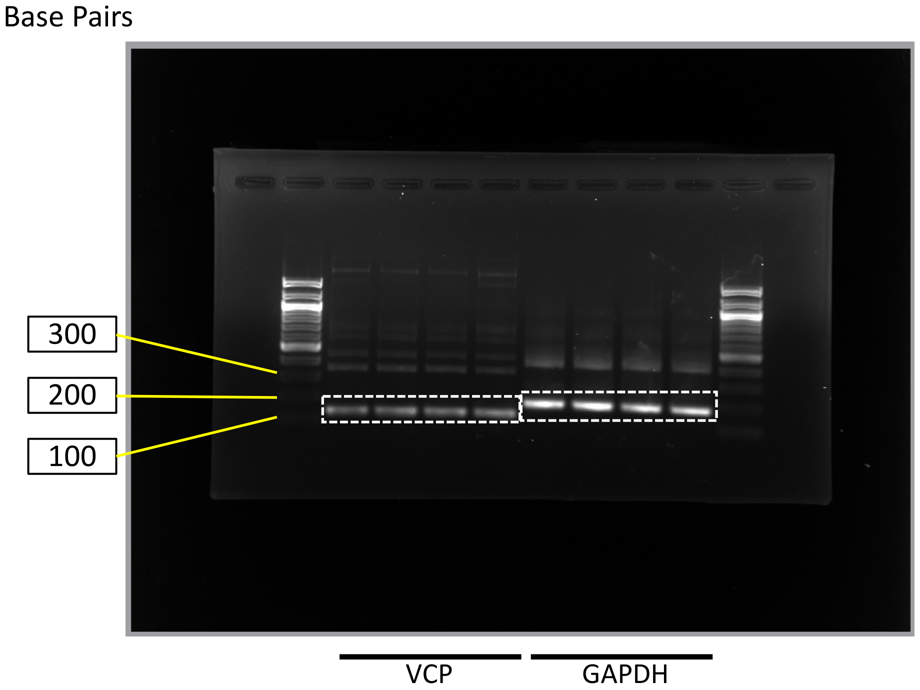

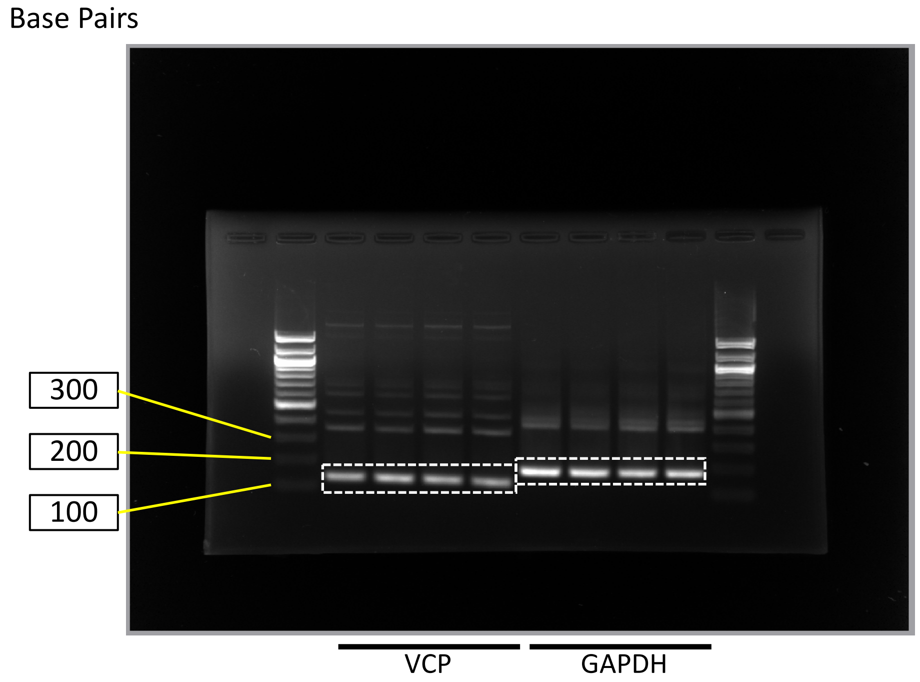

Supplementary Figure S4. The uncropped gels for RT-PCR in **Fig. 1 a** (upper) and **Fig. 3a** (lower). White dashed box represents the cropped image used in main figure.

Supplementary Fig. S5.

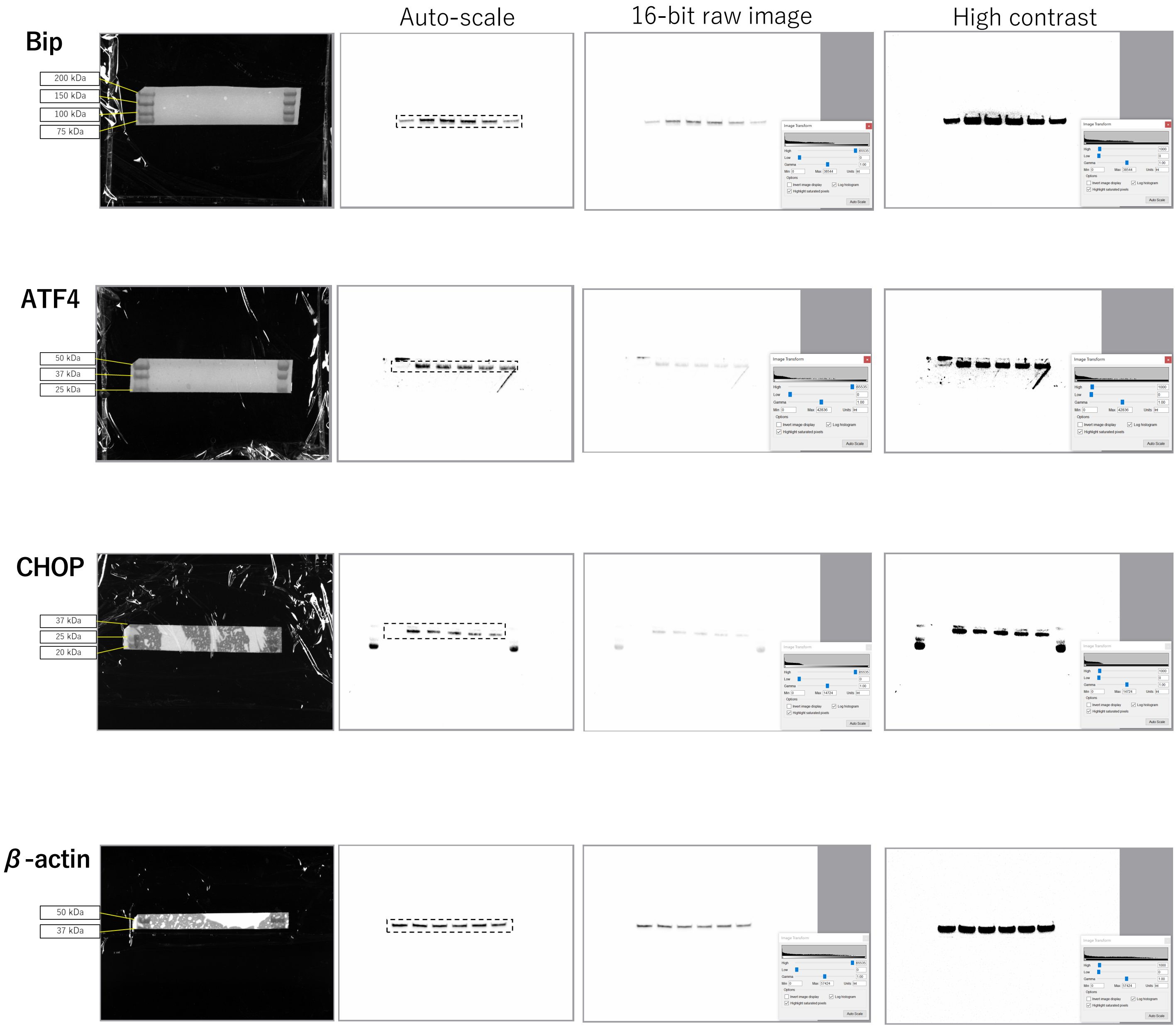

Supplementary Figure S5. The uncropped images of western blots and the corresponding bright field images with protein molecular weight markers used for **Fig. 4a** (left two). An optimal setting for the image was automatically determined by Image Lab software (Bio Rad, CA). Black dashed box represents the cropped image used in **Fig. 4a**. To prove the absence of additional bands, the corresponding raw and excessively high contrast images were also shown (right two). Membranes were cut according to the molecular weight of the targeted protein. The membranes of Bip and ATF4, and the membranes of β-actin and CHOP were cut from the one original membranes, respectively.

Supplementary Fig. S6.

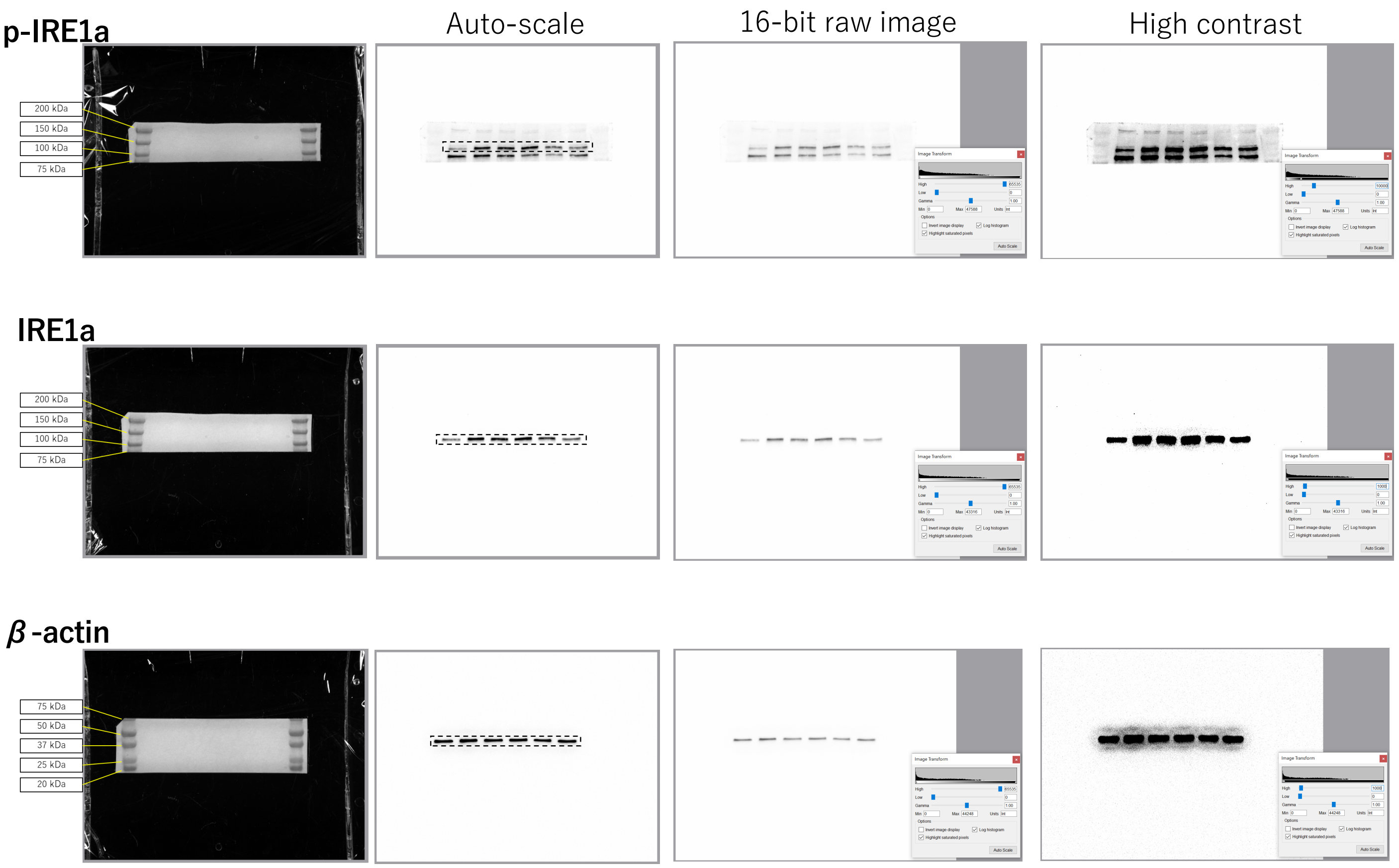

Supplementary Figure S6. The uncropped images of western blots and the corresponding bright field images with protein molecular weight markers used for **Fig. 4e** (left two). An optimal setting for the image was automatically determined by Image Lab software (Bio Rad, CA). Black dashed box represents the cropped image used in **Fig. 4e**. To prove the absence of additional bands, the corresponding raw and excessively high contrast images were also shown (right two). After protein transfer from the gel, the one original membrane were cut to two small membranes by 75kDa (upper half: p-IRE1α and IRE1α, lower half: βactin). After the detection of the p-IRE1α, the bound antibodies were stripped in stripping buffer (Nacalai Tesque, Japan), and the membrane was re-probed with anti-IRE1α antibody.

Supplementary Fig. S7.

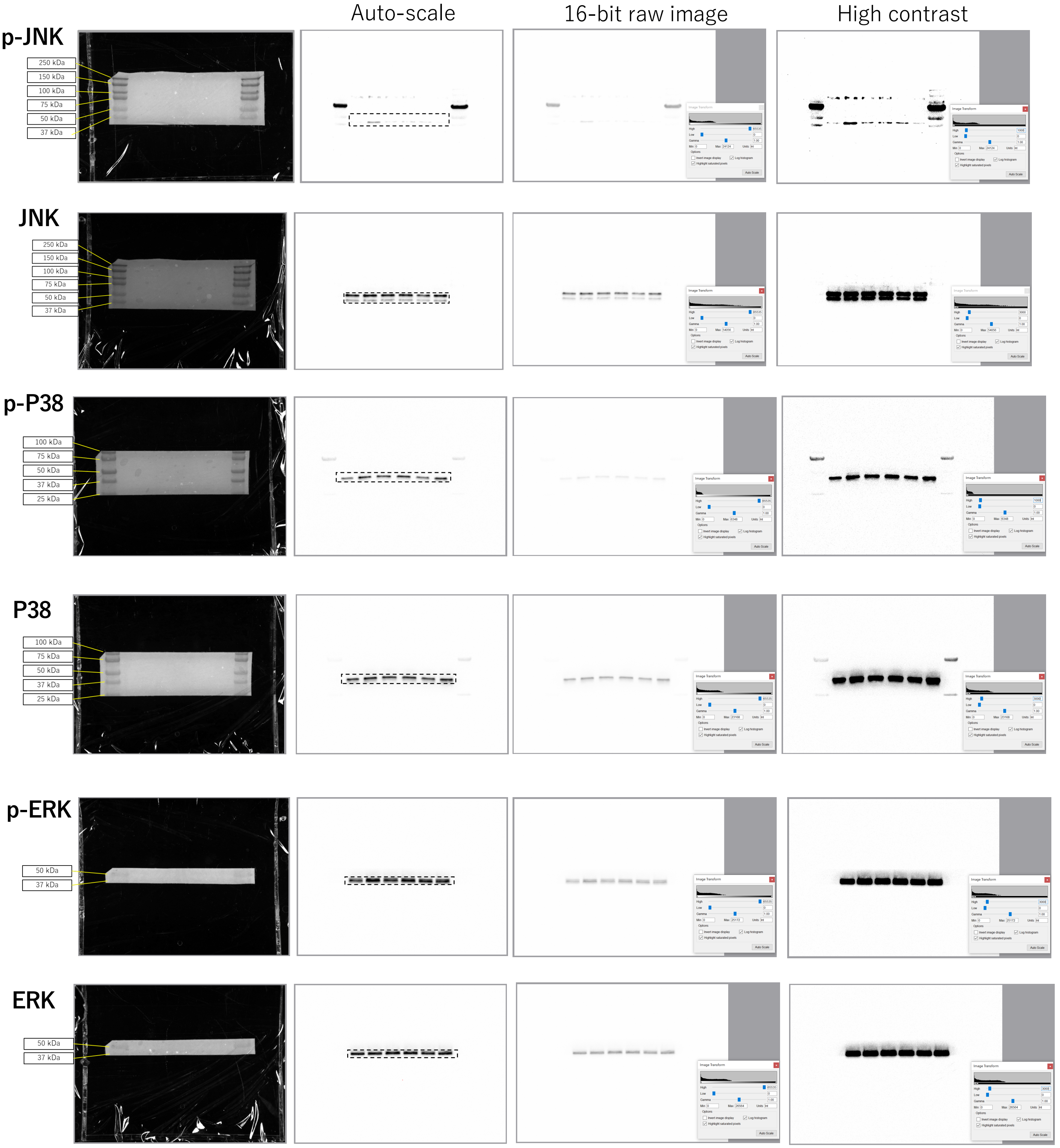

Supplementary Figure S7 . The uncropped images of western blots and the corresponding bright field images with protein molecular weight markers used for **Fig. 5a** (left two). An optimal setting for the image was automatically determined by Image Lab software (Bio Rad, CA). Black dashed box represents the cropped image used in **Fig. 5a**. To prove the absence of additional bands, the corresponding raw and excessively high contrast images were also shown (right two). After probing membranes with the phospho-specific antibodies (p-JNK, p- ERK and p-P38), the bound antibodies were stripped from the membrane by incubating with stripping buffer. Then, the membranes were re-probed with corresponding antibodies (JNK, ERK and P38).

Supplementary Table 1. List of primers used in this paper

Gene	Primer	Sequence (5'→3')	Product size (bp)
Human GAPDH	Forward	TCGGAGTCAACGGATTTGGT	181
	Reverse	TTCCCGTTCTCAGCCTTGAC	
Human MMP-1	Forward	GGGGCTTTGATGTACCCTAGC	142
	Reverse	TGTCACACGCTTTTGGGGTTT	
Human MMP-13	Forward	GTTTGCAGAGCGCTACCTGA	108
	Reverse	GACTGCATTTCTCGGAGCCT	
Human ADAMTS-5	Forward	TCGGGAGGATTTATGTGGGC	172
	Reverse	GGAATCGTCATGGGAGAGGC	
Human IL-1β	Forward	TTCGAGGCACAAGGCACAA	78
	Reverse	TGGCTGCTTCAGACACTTGAG	
Human TNF-α	Forward	CCCATGTTGTAGCAAACCCTC	96
	Reverse	TATCTCTCAGCTCCACGCCA	
Human Sox 9	Forward	GCTCTGGAGACTTCTGAACGA	132
	Reverse	CCGTTCTTCACCGACTTCCT	
Human ACAN	Forward	GCGAGCACTGTAACATAGACAT	127
	Reverse	TCACACAGGTCCCCTTCGTA	
Human col2a1	Forward	GTCCTCTGCGACGACATAATC	178
	Reverse	GTCCTTTGGGTCCTACAATATCC	
Human VCP	Forward	TTCTGGAGCCGATTCAAAAGGTG	136
	Reverse	ATCCATCTTGGGCTGGGACA	
Rat GAPDH	Forward	CGGTGTGAACGGATTTGGC	150
	Reverse	AACTTGCCGTGGGTAGAGTC	
Rat VCP	Forward	TGTAGTTTGGGTGGTGCAGG	147
	Reverse	AAACTGAGGCCCCTTGTCC	

Supplementary Table 2. List of antibodies used in this paper

Antibody	Dilution used for		Host		Type		Supplier	
	WB	IHC	WB	IHC	WB	IHC	WB	IHC
β-actin	1:10000	-	Rabbit	-	Monoclonal	-	CST	-
Bip	1:1000	-	Rabbit	-	Monoclonal	-	CST	-
ATF4	1:1000	-	Rabbit	-	Monoclonal	-	CST	-
CHOP	1:1000	1:800	Mouse	Rabbit	Monoclonal	Monoclonal	CST	Abcam
IRE1α	1:1000	-	Rabbit	-	Monoclonal	-	CST	-
Phospho-IRE1α	1:1000	-	Rabbit	-	Monoclonal	-	Abcam	-
JNK	1:1000	-	Rabbit	-	Monoclonal	-	CST	-
Phospho-JNK	1:1000	-	Rabbit	-	Monoclonal	-	CST	-
P38	1:1000	-	Rabbit	-	Monoclonal	-	CST	-
Phospho-P38	1:1000	-	Rabbit	-	Monoclonal	-	CST	-
ERK	1:1000	-	Rabbit	-	Monoclonal	-	CST	-
Phospho-ERK	1:1000	-	Rabbit	-	Monoclonal	-	CST	-
ADAMTS-5	-	1:800	-	Rabbit	-	Polyclonal	-	Abcam
MMP-13	-	1:1000	-	Rabbit	-	Polyclonal	-	Abcam

WB: western blotting, IHC: immunohistochemistry, CST: Cell Signaling (Danvers, MA, USA), Abcam (Cambridge, UK)
